# 393. Long-Acting Injectable Cabotegravir and Rilpivirine in the Aging HIV Population: Insights from the RELATIVITY Cohort

**DOI:** 10.1093/ofid/ofaf695.131

**Published:** 2026-01-11

**Authors:** Jesús Troya, Carmen Busca, María José Galindo, María Lagarde, Daniel Rodríguez, Miguel Torralba, Alberto Díaz de Santiago, Francisco Fanjul, Adrían Rodríguez, Alfonso Cabello, María José Crussels, Sonia Calzado, María Aguilera, Carmen Hidalgo, Luis Enrique Morano, David Vinuesa, Karenina Antelo, Enrique Bernal, Rosa María Martínez, Noemí Cabello, Juan Tiraboshi, María del Carmen Montero, María Jesús Vivancos, Cristina Díez, Guillermo Soria, Desiré Pérez, Laura Gisbert, Alberto Romero, Rebeca Cabo, Josefa Francisca Soler, Maria Antonia Sepúlveda, Cristina Escrich, Antonio Jesús Sánchez, Noelia Ruiz, Eva María Ferreira, Beatriz Valentín, Javier Lopéz-Nieto, Jara Llenas, Albert Gómez, Juan Emilio Losa, Jose Fernando Lluch, José Sanz, Sergio Padila, Hadrián Pernas, Juan José Corte, Ester Sáez, María Ángeles Garcinuño, Miriam Estébanez, María de Mar García, Luis Buzón-Martín

**Affiliations:** Hospital Universitario Infanta Leonor, Madrid, Madrid, Spain; Hospital Universitario La Paz, Madrid, Madrid, Spain; Hospital Clínico de Valencia, Valencia, Comunidad Valenciana, Spain; Hospital Universitario 12 de Octubre, Madrid, Madrid, Spain; Hospital Universitario de Canarias, Santa Cruz de Tenerife, Canarias, Spain; Hospital Universitario de Guadalajara, Guadalajara, Madrid, Spain; Hospital Universitario Puerta de Hierro, Madrid, Madrid, Spain; Hospital Universitario Son Espases, Palma de. Mallorca, Islas Baleares, Spain; Hospital Universitario Son Llatzer, Palma de mallorca, Islas Baleares, Spain; Hospital Universitario Fundación Jiménez Díaz, Madrid, Madrid, Spain; Hospital Clí­nico Universitario Lozano Blesa, Zaragoza, Castilla y Leon, Spain; Hospital Universitario Parc Taulí, Sabadell, Catalonia, Spain; Hospital Universitario de la Princesa, Madrid, Madrid, Spain; Hospital Universitario Virgen de las Nieves, Granada, Andalucia, Spain; Hospital Universitario Álvaro Cunqueiro, Vigo, Galicia, Spain; Hospital Clí­nico San Cecilio, Granada, Andalucia, Spain; Hospital de Denia Marina Salud, Alicante, Comunidad Valenciana, Spain; Reina Sofía General University Hospital, Murcia, Murcia, Spain; Hospital Universitario Miguel Servet, Zaragoza, Aragon, Spain; Hospital Clínico Universitario de Madrid, Madrid, Madrid, Spain; Hospital Universitario de Bellvitge, Bellvitge, Catalonia, Spain; Hospital Universitario de Torrejón, Torrejón, Madrid, Spain; Hospital Universitario Ramón y Cajal, Madrid, Madrid, Spain; Hospital General Universitario Gregorio Marañón, Madrid, Madrid, Spain; Hospital Universitario de Fuenlabrada, Fuenlabrada, Madrid, Spain; Hospital Universitario San Agustín, Avilés, Asturias, Spain; Hospital Universitario Mutua de Terrassa, Barcelona, Catalonia, Spain; Hospital Universitario de Puerto Real, Cádiz, Andalucia, Spain; Hospital Universitario Central de Asturias, Oviedo, Asturias, Spain; Hospital Universitario de Cabueñes, Cabueñes, Asturias, Spain; Hospital Universitario de Toledo, Toledo, Castilla-La Mancha, Spain; Hospital Verge de la Cinta de Tortosa, Tarragona, Catalonia, Spain; Hospital General Universitario Morales Meseguer, Murcia, Murcia, Spain; Hospital Universitario Marqués de Valdecilla, Santander, Cantabria, Spain; Complejo Asistencial de Segovia, Segovia, Castilla y Leon, Spain; Hospital Rí­o Hortega de Valladolid, Valladolid, Castilla y Leon, Spain; Hospital de Viladecans, Viladecans, Catalonia, Spain; Hospital de la Vega Baja, Alicante, Comunidad Valenciana, Spain; Hospital de Santa Caterina de Salt, Girona, Catalonia, Spain; Hospital Universitario de Alcorcón (Madrid), Alcorcón, Madrid, Spain; Hospital Universitario Doctor José Molina Orosa, Las Palmas, Canarias, Spain; Hospital Universitario Prí­ncipe de Asturias, Alcalá de Henares, Madrid, Spain; Hospital General Universitario de Elche, Elche, Comunidad Valenciana, Spain; Complejo Hospitalario Universitario de Pontevedra, Pontevedra, Galicia, Spain; Hospital de Jove, Alicante, Comunidad Valenciana, Spain; Hospital de Jove, Alicante, Comunidad Valenciana, Spain; Complejo Asistencial de Ávila, Ávila, Castilla y Leon, Spain; Hospital Central de la Defensa Gómez Ulla, Madrid, Madrid, Spain; Hospital de Vinalopó, Vinalopó, Comunidad Valenciana, Spain; Hospital Universitario de Burgos, burgos, Castilla y Leon, Spain

## Abstract

**Background:**

Long-acting injectable cabotegravir and rilpivirine (LAI CAB+RPV) has become a standard treatment option for people living with HIV (PLWH), offering high efficacy, safety, and convenience. However, data from clinical trials and real-world cohorts involving people over 60 - an important and growing population with physiological differences and emerging comorbidities - remain scarce.

Virological Efficacy Over Time by Age Group Among PLWH on LAI CAB+RPV.

**Figure:**
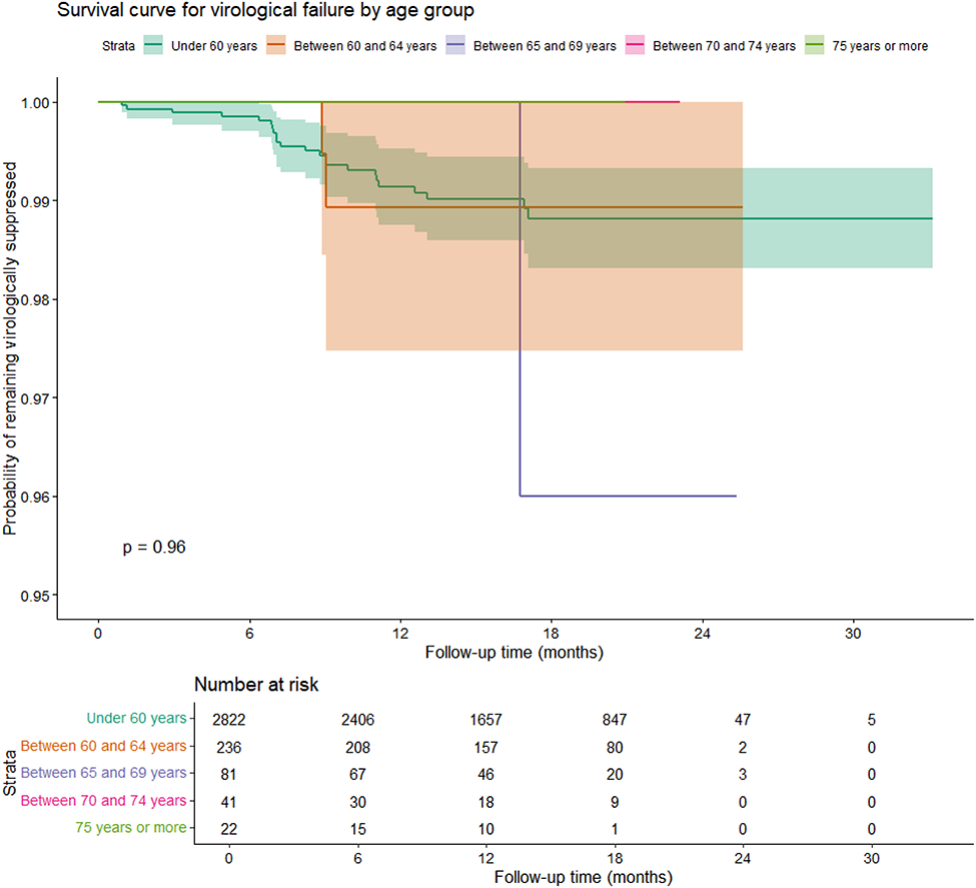

**Methods:**

We conducted a multicenter, retro-prospective study within the Spanish RELATIVITY cohort, analyzing virologically suppressed PLWH aged ≥60 years who transitioned to LAI CAB+RPV. We described this population and evaluated factors associated with virological outcomes using Kaplan–Meier analysis and Cox proportional hazards models.

**Results:**

This substudy included 380 PLWH from 58 Spanish hospitals (11.8% of the RELATIVITY cohort). The median age was 63 years (IQR, 61–67); 78% were male, and 90.3% were Spanish nationals. Comorbidities were present in 79.5% of participants, with dyslipidemia (51.6%), hypertension (36.3%), and osteoporosis (15%) being the most common. The median time on ART prior to switching was 18 years (IQR, 11–25), and the median duration of sustained viral suppression was 11 years (IQR, 6.3–17). The main reasons for switching to LAI CAB+RPV were to improve comfort or quality of life (51.7% in those < 60 years vs. 45.8% in those ≥60 years; p = 0.034) and treatment simplification (23.6% vs. 25.5%; p = 0.436). Virological success exceeded 96.5% across all age groups, with a low virological failure rate observed in the older population (0.8%; p = 0.844). Age was not significantly associated with virological failure in either Kaplan–Meier or Cox proportional hazards analyses (HR, 0.996; 95% CI, 0.962–1.031; p = 0.814). Discontinuation rates for any cause were similar between groups (5.2% vs. 5.8%; p = 0.873).

No statistically significant differences were found in adverse event rates (0.8% vs. 1.6%; p = 0.335), including injection site reactions (1.4% vs. 1.3%; p = 0.801).

**Conclusion:**

In a real-world setting, LAI CAB+RPV is a viable and effective treatment option for people over 60 living with long-standing HIV infection and multiple comorbidities. The regimen demonstrated excellent virological control and was well-tolerated across all age groups.

**Disclosures:**

All Authors: No reported disclosures

